# Elevated expression of LPCAT1 predicts a poor prognosis and is correlated with the tumour microenvironment in endometrial cancer

**DOI:** 10.1186/s12935-021-01965-1

**Published:** 2021-05-20

**Authors:** Tianyi Zhao, Yifang Zhang, Xiaohong Ma, Lina Wei, Yixin Hou, Rui Sun, Jie Jiang

**Affiliations:** 1 Department of Gynecology and Obstetrics, Qilu Hospital, Cheeloo College of Medicine, Shandong University, 107 West Wenhua Rd, Jinan, 250012 Shandong Peoples Republic of China; 2 Department of Gynecology and Obstetrics, The Second Affiliated Hospital of Shandong First Medical University, 366 Taishan Road, Taian, 271000 Shandong Peoples Republic of China

**Keywords:** Glycerophospholipids, Endometrial cancer, LPCAT1, Tumor microenvironment

## Abstract

**Background:**

Endometrial cancer (EC) is one of the three malignant reproductive tumours that threaten womens lives and health. Glycerophospholipids (GPLs) are important bioactive lipids involved in various physiological and pathological processes, including cancer. Immune infiltration of the tumour microenvironment (TME) is positively associated with the overall survival in EC. Exploring GPL-related factors associated with the TME in endometrial cancer can aid in the prognosis of patients and provide new therapeutic targets.

**Methods:**

Differentially expressed GPL-related genes were identified from TCGA-UCEC datasets and the Molecular Signatures Database (MSigDB). Univariate Cox regression analysis was used to select GPL-related genes with prognostic value. The Random forest algorithm, LASSO algorithm and PPI network were used to identify critical genes. ESTIMATEScore was calculated to identify genes associated with the TME. Then, differentiation analysis and survival analysis of LPCAT1 were performed based on TCGA datasets. GSE17025 and immunohistochemistry (IHC) verified the results of the differentiation analysis. An MTT assay was then conducted to determine the proliferation of EC cells. GO and KEGG enrichment analyses were performed to explore the underlying mechanism of LPCAT1. In addition, we used the ssGSEA algorithm to explore the correlation between LPCAT1 and cancer immune infiltrates.

**Results:**

Twenty-three differentially expressed GPL-related genes were identified, and eleven prognostic genes were selected by univariate Cox regression analysis. Four significant genes were identified by two different algorithms and the PPI network. Only LPCAT1 was significantly correlated with the tumour microenvironment. Then, we found that LPCAT1 was highly expressed in tumour samples compared with that in normal tissues, and lower survival rates were observed in the groups with high LPCAT1 expression. Silencing of LPCAT1 inhibited the proliferation of EC cells. Moreover, the expression of LPCAT1 was positively correlated with the histologic grades and types. The ROC curve indicated that LPCAT1 had good prognostic accuracy. Receptor ligand activity, pattern specification process, regionalization, anterior/posterior pattern specification and salivary secretion pathways were enriched as potential targets of LPCAT1. By using the ssGSEA algorithm, fifteen kinds of tumor-infiltrating cells (TICs) were found to be correlated with LPCAT1 expression.

**Conclusion:**

These findings suggested that LPCAT1 may act as a valuable prognostic biomarker and be correlated with immune infiltrates in endometrial cancer, which may provide novel therapy options for and improved treatment of EC.

**Supplementary Information:**

The online version contains supplementary material available at 10.1186/s12935-021-01965-1.

## Introduction

Endometrial cancer (EC) is one of the most prevalent gynaecological cancers worldwide, with an incidence of approximately 4.5% in female cancer cases, and over 410,000 new cases were diagnosed in 2020 [1]. The treatment of endometrial cancer is mainly surgical treatment complementary with chemotherapy, radiotherapy and hormone therapy. Other options include immunotherapy, which mobilizes the immune system against cancer, and targeted therapy with drugs that attack specific weaknesses in cancer cells. Despite the great advances in medical devices and treatment in recent years, the mortality of EC has increased in the past several years, and treatment effects are poor for patients with advanced and specific subtypes [2]. Endometrial cancer often occurs in women with metabolic disorders, including diabetes, hypertension and obesity [3]. A full understanding of metabolic alterations could contribute to early diagnosis, timely treatment and the development of new therapeutic targets.

Glycerophospholipids (GPLs) are fundamental components of biomembrane systems. GPL metabolism is one of the most important components of maintaining body homeostasis. Enhanced synthesis of GPLs provides a sufficient membrane structure for rapid cell proliferation and serves as a source of energy supply under conditions of nutrient deficiency [4]. The dynamic changes in the substrates and products of GPL metabolism, such as phospholipids and free fatty acids, have profound consequences on intracellular signal transduction. The composition of fatty acyl chains in individual GPLs contributes to specific biophysical properties of the cell membrane and influences a variety of cellular processes [5]. Accumulating evidence has shown that GPL-related genes that regulate GPL synthesis and remodelling are involved in the development of cancer. Phospholipase A2 enzymes (PLA2s) has been found to be significantly overexpressed in various tumour tissues, including colorectal cancer, prostate cancer and gastric cancer, and patients with higher PLA2 expression have a poor prognosis [6]. A study by Wang et al. demonstrated that phospholipid remodelling caused by lysophosphatidylcholine acyltransferase 3 (LPCAT3) deletion promoted the proliferation and division of small intestinal stem cells [7]. Moreover, Trousil et al. found that upregulation of choline kinase (CHKA) was responsible for alterations of choline phospholipid metabolism in EC and validated abnormal choline biochemistry as a biomarker for EC [8]. Although attention has been given to the role of GPL-related genes in EC progression, their effects have not been well characterized.

The tumour microenvironment is an ecosystem composed of mesenchymal cells, immune cells, extracellular matrix (ECM) molecules and inflammatory mediators that have vital impacts on tumour progression and clinical outcomes [9]. The structural components of the TME contain not only cancer cells but also recruited immune cells and resident stromal cells. Studies have shown that immune and stromal scores are positively correlated with the clinical characteristics and outcomes of EC [10]. Moreover, several genes related to the EC immune environment can be used to predict prognosis [11]. Lipid metabolites are important mediators that affect the tumour microenvironment. A recent study showed that the GPL metabolism levels influenced the effect of PD-1 antibodies by changing the components of the tumour microenvironment (TME) in colorectal cancer [12]. However, whether GPL-related genes influence the EC microenvironment and clinical outcomes is largely unknown.

Here, our results indicated that the GPL-related gene LPCAT1 had effects on the endometrial cancer TME and clinical outcomes of EC patients, which might provide a novel prognostic biomarker and immunotherapy target for EC.

## Materials and methods

### Generation of GPLs-related DEGs

Level 3 transcriptome RNA-seq data and the corresponding clinical data, which contained 552 endometrial cancer cases and 35 normal control cases, were downloaded from The Cancer Genome Atlas (TCGA) (https://portal.gdc.cancer.gov/) using the TCGAbiolinks package of R-software (version 4.0.3, the same below). GPL-related gene sets were obtained from the Molecular Signatures Database (MSigDB) on the gene set enrichment analysis (GSEA) website. Analysis of differentially expressed genes (DEGs) was performed using the limma [13] package at a corrected p<0.05 and | log_2_FC |1. A Venn diagrams tool (http://bioinformatics.psb.ugent.be/webtools/Venn/) was used to identify the intersecting genes between upregulated and downregulated DEGs and GPL-related gene sets and were selected for further analysis.

### Identification of the core prognostic GPLs-related genes

Based on the expression of GPL-related DEGs, univariate Cox proportional hazards regression analysis was performed to identify genes associated with endometrial cancer prognosis, and p values<0.05 were regarded as significant. Then, survival-related genes were subjected to least absolute shrinkage and selection operator (LASSO) penalty Cox regression analysis with the R package glmnet [14] to eliminate false positives caused by over-fitting. Tenfold cross-validation was used to tune the parameter (lambda) selection in the LASSO model. lambda.min was chosen as the cut-off point because it provides the minimum mean cross-validated error, and genes with the highest lambda values were selected for further analysis. The patients were randomly divided into a training set and testing set according at a ratio of 7:3. Random forests built for candidate gene selection from the training set and were verified in the testing set by the randomForest R package with 500 trees and default settings [15]. To understand protein interactions, we constructed a proteinprotein intersection (PPI) network by STRING (V11) [16] with high confidence (0.65). Overlapping genes in the PPI network, LASSO and random forest algorithms were selected.

### Generation of ImmuneScore, StromalScore, and ESTIMATEScore

The ESTIMATE algorithm in R language loaded with the estimate package [17] was used to estimate the ratio of the immune-stromal components in the TME for each sample using the following scores: ImmuneScore, StromalScore, and ESTIMATEScore. These scores are positively correlated with the ratio of immune components, stromal components, and the sum of both components, which means that the higher the scores are, the greater the proportion of the corresponding components in the TME.

### Survival and expression analysis of LPCAT1

Box plots using disease state (tumour or normal) as a variable were graphed to visualize the differential expression of LPCAT1. Expression data for LPCAT1 in the GSE17025 data set were downloaded from the GEO database (https://www.ncbi.nlm.nih.gov/geo/) to verify its differential expression between tumour and normal tissues. Box plots using pathological type and cancer grade as variables were analysed to compare the LPCAT1 expression in cancers with different pathological types and grades. KaplanMeier analysis was conducted to evaluate the relationship between LPCAT1 expression and survival rate using the survival and survminer R package, including overall survival (OS), disease-free interval (DFI) and progress-free survival (PFS). In addition, receiver operating characteristic (ROC) curve analysis using the pROC R package confirmed the LPCAT1s predictive capacity.

### Enrichment analysis

All the patients were divided into two groups according to the median expression of LPCAT1, and DEG analysis was performed using the limma package at a corrected p<0.05 and | log_2_FC |1. The resulting data were used to generate volcano plots using the ggplot2 package in R. GO and KEGG enrichment analyses of 379 DEGs were performed using the clusterProfiler, enrichplot, and ggplot2 packages in R. Only terms with both *p*- and *q*-values<0.05 were considered significantly enriched. A PPI network was used to show the interactions of each protein.

### Immunohistochemistry analysis

Pathological specimens from 25 EC tissue samples and 11 normal tissue samples were obtained from Qilu Hospital of Shandong University. The tissue samples were fixed with 4% paraformaldehyde and then embedded in paraffin. After being cut into 4m sections, they were deparaffinized and rehydrated with xylene and a graded concentration of ethanol. Tris/EDTA buffer was used to perform heat-mediated antigen retrieval. Then, the sections were incubated in 3% H_2_O_2_ to reduce endogenous peroxidase activity and blocked in 10% goat serum for 30min to reduce nonspecific antigens. The sections were incubated with a rabbit monoclonal anti-LPCAT1 antibody (1:2000 dilution, Abcam ab214034) overnight at 4C, followed by incubation with a biotin-labelled secondary goat anti-rabbit antibody and horseradish enzyme-labelled streptomycin at 37C for 30min. DAB reagent was used to detect positive signals. The integrated optical density (IOD) of each section was calculated using Image-Pro Plus software 6.0 (Media Cybernetics, USA) by two independent blinded investigators randomly.

### TICs profile

To explore the differences in immune cell subtypes, single sample gene set enrichment (ssGSEA) analysis using the GSVA package was conducted to estimate the TIC abundance profiles in all tumour samples. The hallmark gene sets for 28 immune cell types were downloaded from a recent publication [18]. The enrichment scores were used to perform subsequent correlation analysis. The MannWhitney U test was used to compare differences in immune cell subtypes between the high LPCAT1 and low LPCAT1 groups.

### Cell culture and transfection

The human endometrial cancer cell lines Ishikawa and HEC-1A were purchased from the ZhongQiaoXinZhou Biotechnology Co., Ltd. (Shanghai, China). The human endometrial cancer cell line RL95-2 was purchased from the Shanghai Cell Bank (Shanghai, China). Ishikawa, HEC-1A and RL95-2 cells were cultured in RPMI 1640 medium (BI, Israel), McCoys 5A medium (Macgene, Beijing, China) and DMEM/F12 medium (BI, Israel) supplemented with 10% foetal bovine serum (FBS; HyClone), streptomycin (100g/mL) and penicillin (100 U/mL) (Thermo Scientific, Waltham, MA, USA). Cells were cultured at 37C in the presence of a humidified atmosphere with 5% CO_2_. All cell lines were free of mycoplasma and were verified by short tandem repeats (STRs). LPCAT1-specific small interfering RNA (siRNA) and a control scrambled siRNA used as a negative control (NC) were designed and synthesized by GenePharma. The nucleotide sequences of the siRNAs were as follows: si-LPCAT1 (sense 5-CCAUGACGAUGUCCUCCAUTT-3, antisense 5-AUGGAGGACAUCGUCAUGGTT-3) and si-NC (sense 5-UUCUCCGAACGUGUCACGUTT-3, antisense 5-ACGUGACACGUUCGGAGAATT-3). Cells were transfected using Lipofectamine 3000 Reagent (Invitrogen, CA, USA) according to the manufacturers protocol at 50% confluency.

### Western blotting

Cells were washed with ice-cold 1PBS 3 times, and total cell lysates were prepared using RIPA lysis buffer with 1% NaF and 1% phenylmethylsulfonyl fluoride (PMSF) for 30min. The concentrations of proteins were determined with a BCA protein assay kit (Beyotime, Beijing, China). Denatured proteins were separated on a 12% SDSpolyacrylamide gel and then transferred to polyvinylidene fluoride membranes. After blocking with 5% defatted milk, membranes were blocked overnight at 4C with an anti-LPCAT1 primary antibody (ab214034, 1:1000 dilution, Abcam, Cambridge, MA, USA). Next, the membranes were incubated with a secondary antibody, and the bands were detected using enhanced chemiluminescence (Thermo Fisher Scientific Inc., MA, USA). The -actin band acted as a control.

### MTT assay

After transfection with si-NC or si-LPCAT1, Ishikawa, HEC-1A and RL95-2 cells were seeded at a concentration of 3000 cells/100l in 96-well plates overnight to adhere. Twenty microlitres of 3-(4,5-dimethylthiazol-2-yl)-2,5-diphenyltetrazolium bromide (MTT) solution (5mg/ml) was added to each well at a fixed time from Day 1 to Day 5. After incubation at 37C for 4h in the dark, the supernatants were discarded, and 100l dimethyl sulfoxide (DMSO) was added to the wells for 10min to dissolve the formazan crystals that formed. Absorbance was then read at 490nm using a microplate absorbance reader.

## Results

**Fig. 1:**
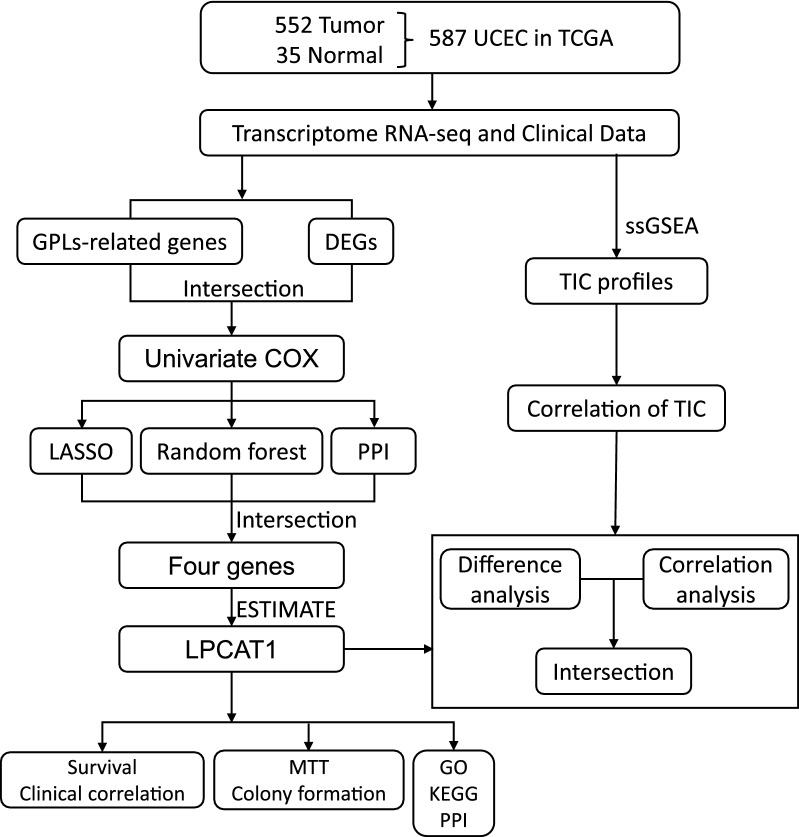
Flow chart of data collection and analysis

The flow chart of this study is shown in Fig.1. A total of 552 UCEC patients from the TCGA-UCEC cohort were finally enrolled. The detailed clinical characteristics (Additional file 4: Table S2) including clinical stage, primary therapy outcome, age, BMI, histological type, histologic grade, overall survival event, progression-free interval, diabetes, and tumour invasion, of the included patients are summarized in Table 1.

**Table 1:** Clinical pathological parameters of the UCEC patients included in this study

Characteristic	Level	Overall
n		552
Clinical stage, n (%)	Stage I	342 (62%)
	Stage II	51 (9.2%)
	Stage III	130 (23.6%)
	Stage IV	29 (5.3%)
Primary therapy outcome, n (%)	PD	20 (4.2%)
	SD	6 (1.2%)
	PR	12 (2.5%)
	CR	442 (92.1%)
Age, n (%)	60	206 (37.5%)
	>60	343 (62.5%)
BMI, n (%)	30	212 (40.8%)
	>30	307 (59.2%)
Histological type, n (%)	Endometrioid	410 (74.3%)
	Mixed	24 (4.3%)
	Serous	118 (21.4%)
Histologic grade, n (%)	G1	98 (18.1%)
	G2	120 (22.2%)
	G3	323 (59.7%)
OS event, n (%)	Alive	458 (83%)
	Dead	94 (17%)
PFI event, n (%)	Alive	423 (76.6%)
	Dead	129 (23.4%)
Diabetes, n (%)	No	328 (72.7%)
	Yes	123 (27.3%)
Tumour invasion (%), n (%)	<50	259 (54.6%)
	50	215 (45.4%)

### Identification of prognostic GPL-related differentially expressed genes (DEGs)

**Fig. 2:**
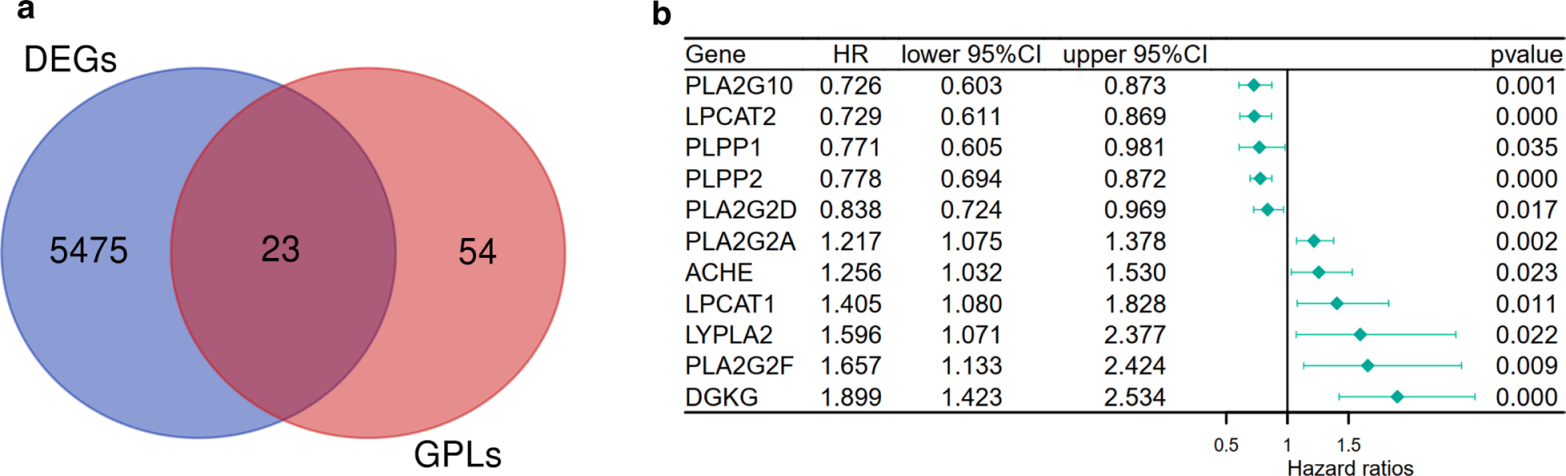
Identification of prognostic GPL-related differentially expressed genes in UCEC patients. **a** Venn plots showing intersecting genes shared by DEGs between tumour and adjacent normal tissues and GPL-related genes. **b** Univariate Cox regression analysis of the expression of 23 GPL-related genes and OS was performed and shown on a forest plot, listing 11 significant factors with *p*<0.05

To identify the DEGs between tumour tissues and normal tissues in the TCGA-UCEC cohort, differential analysis using the limma package was performed. Twenty-four GPL-related genes were found to be differentially expressed between tumour tissues and normal tissues (Fig.2a). Univariate Cox regression analysis to determine the survival of UCEC patients was performed to identify the prognostic factors among 23 DEGs, and 11 genes were selected (Fig.2b).

### Intersection of the random forest model and LASSO Cox model and analysis of the PPI network

**Fig. 3:**
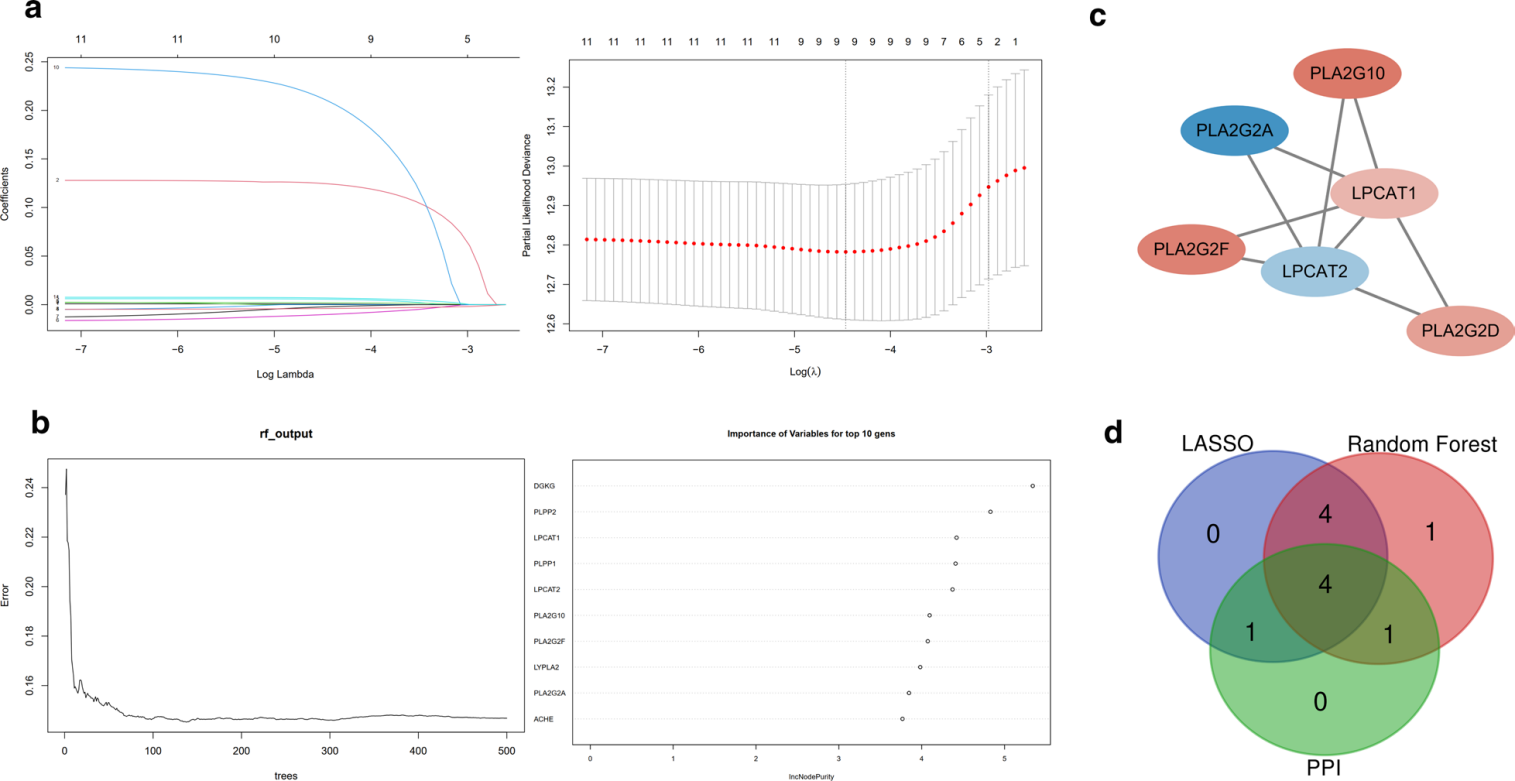
LASSO penalized Cox model, random forest model and PPI analysis for selecting core GPL-related genes. **a** Nine key GPL-related genes were predicted based on ridge regression and LASSO. **b** The top ten genes were selected by the random forest algorithm. **c** The PPI network downloaded from the STRING database was constructed with nodes with interaction confidence values >0.65. **d** Venn plot displaying four core GPL-related intersecting genes according to the LASSO algorithm, random forest algorithm and PPI analysis

To further identify core GPL-related genes, we used two different machine learning algorithms, the LASSO algorithm and random forest algorithm. By the LASSO algorithm, 9 out of 11 genes were identified (Fig.3a). The areas under the ROC curve (AUCs) (Additional file 1: Figure S1a, AUC:0.698) and risk score were calculated based on the coefficients determined by the LASSO algorithm. The risk scores of patients who died of disease were significantly higher than those of patients who survived (Additional file 1: Figure S1b). Simultaneously, the top 10 genes out of 11 were confirmed by the random forest algorithm (Fig.3b), and these genes, to a certain extent, can predict the prognosis of UCEC patients not only in the training set (Additional file 2: Figure S2a) but also in the testing set (Additional file 2: Figure S2b). Additionally, we constructed a PPI network based on the STRING database using Cytoscape software [National Institute of General Medical Sciences (NIGMS) USA] to explore the underlying mechanism (Fig.3c). Then, the LASSO algorithm was cross-analysed with the top 10 genes ranked by the random forest algorithm, and the leading nodes in the PPI network and four factors, PLA2G2F, PLA2G2A, LPCAT2 and LPCAT1, were found to overlap in the above analysis (Fig.3d).

### The expression of LPCAT1 is associated with the EC microenvironment

**Fig. 4:**
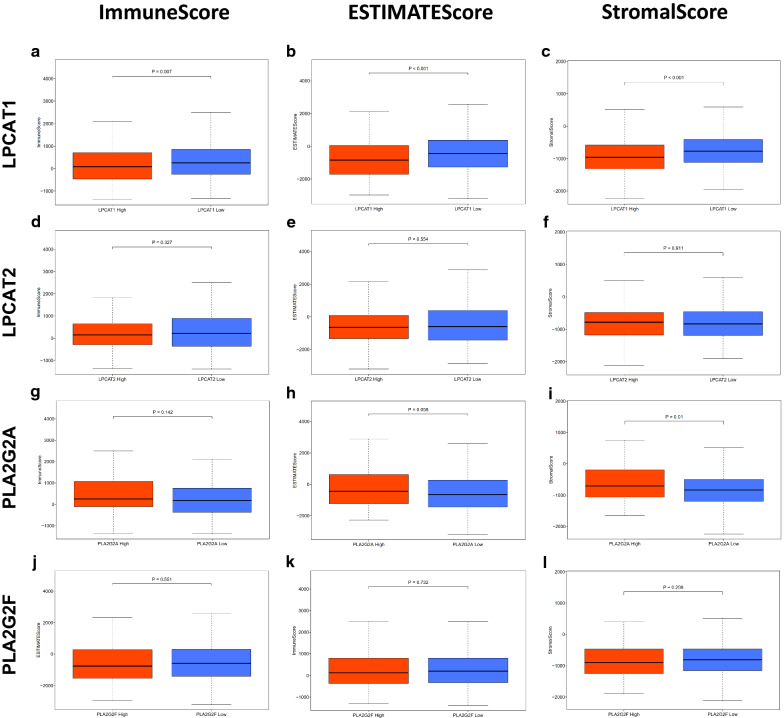
Correlation of ImmuneScore, StromalScore and ESTIMATEScore of each UCEC patient with the expression of four core GPL-related genes. **ac** Distribution of ImmuneScore, StromalScore and ESTIMATEScore in terms of LPCAT1 expression (p=0.007, p<0.001, p<0.001, respectively, by Wilcoxon rank sum test). **df** Distribution of the three scores in terms of LPCAT2 expression (p=0.327, 0.554, 0.911 for ImmuneScore, StromalScore and ESTIMATEScore, respectively, by Wilcoxon rank sum test). **gi** Distribution of the scores in terms of PLA2G2A expression (p=0.142, 0.058, 0.01, for ImmuneScore, StromalScore, and ESTIMATEScore separately by Wilcoxon rank sum test). **jl** Distribution of the scores in terms of PLA2G2F expression (p=0.551, 0.732, 0.298, respectively, by Wilcoxon rank sum test)

To determine the relationship between the proportion of immune and stromal components and the expression of core GPL-related genes, data from 552 EC cases and 19618 RNAs extracted from RNA-seq data from ENSEMBL Genomes (hg38) were analysed in this study. All cases were divided into the high-expression group and low-expression group according to the median expression of the above four core GPL-related genes. ImmuneScore, ESTIMATEScore and StromalScore were predicted by expression profile data using the ESTIMATE R package (Additional file 3: Table S1). We observed no significant differences in ImmuneScore, ESTIMATEScore or StromalScore between the high-expression group and the low-expression group for LPCAT2 (Fig.4df) and PLA2G2F (Fig.4jl). Although the expression of PLA2G2A had a significant correlation with StromalScore (Fig.4i), there was no significant correlation between PLA2G2A expression and ImmuneScore or ESTIMATEScore (Fig.4g, h). However, ImmuneScore, ESTIMATEScore and StromalScore of LPCAT1 in the low-expression group were significantly higher than those in the high-expression group (Fig.4ac). These results suggested that the expression of LPCAT1 was associated with the structural components of the EC microenvironment.

### Relationships between LPCAT1 expression and survival, grade, and histological types in UCEC patients

**Fig. 5:**
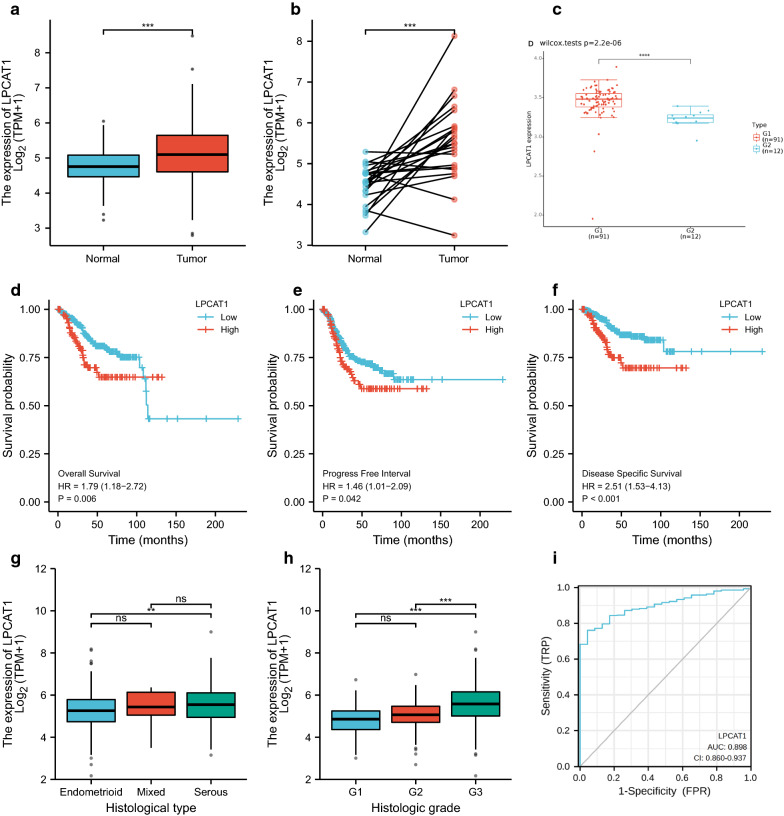
Differentiated expression of LPCAT1 in tumour and normal samples and the correlation of LPCAT1 expression with the survival and clinicopathological characteristics of TCGA-UCEC patients. **a** Differentiated expression of LPCAT1 in the normal and tumour samples. Analyses were performed across all normal and tumour samples with ^***^p<0.001 by Wilcoxon rank sum test. **b** Paired differentiation analysis for the expression of LPCAT1 in normal and tumour samples derived from the same patient (^***^p<0.001 by the Wilcoxon rank sum test). **c** Differentiated expression of LPCAT1 in the normal and tumour samples in GSE17025 (^****^p<0.0001 by the Wilcoxon rank sum test). **df** Overall survival, progression-free interval and disease-specific survival analysis of UCEC patients with different LPCAT1 expression levels. Patients were labelled as high expression or low expression according to the optimal cut-off value (minimum p-value). p=0.006, 0.042, 0.036 by log-rank test. **g**, **h** The correlation of LPCAT1 expression with histologic grade and histological type. The Wilcoxon rank sum or KruskalWallis rank sum test served as the statistical significance test. **i** ROC curve for judging the accuracy of LPCAT1(AUC=0.898)

According to TCGA analysis, we found that LPCAT1 expression in endometrial cancer tissues was significantly higher than that in normal tissues (Fig.5a). Additionally, the expression level of LPCAT1 in tumour tissues was also significantly higher than that in paired normal tissues (Fig.5b). The same finding was verified in GSE17025 (Fig.5c). From the KaplanMeier curve based on TCGA data, lower overall survival (OS), progression-free interval (PFI), and disease-specific survival (DSS) rates were found in patients in the LPCAT1 high expression group than in patients in the LPCAT1 low expression group (Fig.5df). As shown in Fig.5, LPCAT1 expression showed a positive correlation with grade (Fig.5g). The expression of LPCAT1 in serous EC tissues was significantly higher than that in endometrioid EC tissues (Fig.5h). The AUC value was 0.898, indicating a good accuracy of the prognostic prediction value of LPCAT1 (Fig.5i).

### Silencing the expression of LPCAT1 inhibits the proliferation of endometrial cancer cells

**Fig. 6:**
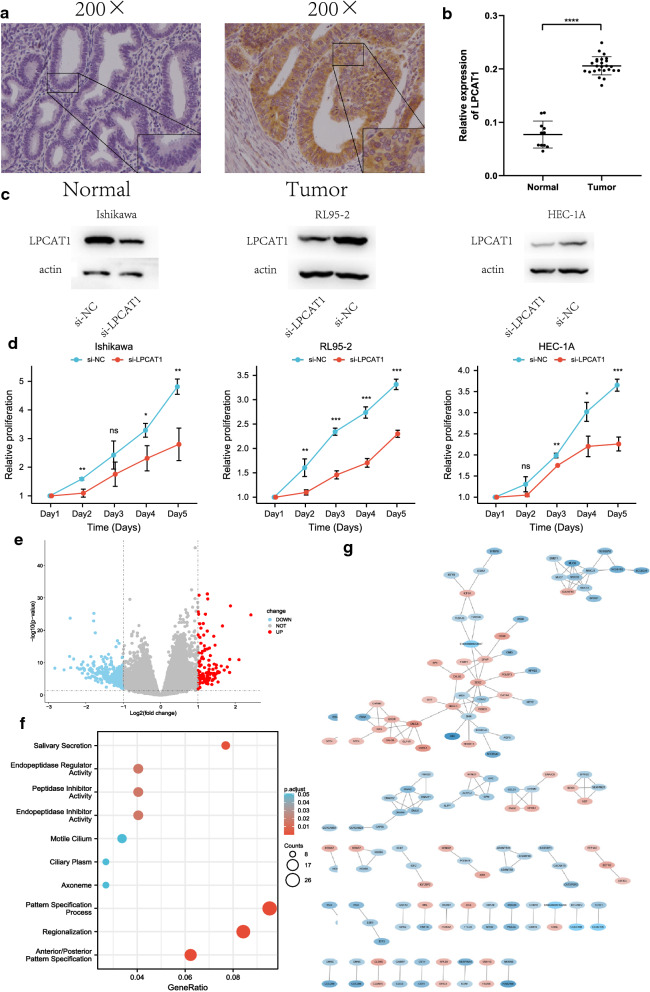
Silencing the expression of LPCAT1 inhibits the proliferation of endometrial cancer cells. Volcano plot, proteinprotein interaction and enrichment analyses of GO and KEGG for DEGs between the high-expression group and low-expression group of LPCAT1. **a** Representative image of LPCAT1 expression in endometrial cancer tissues and normal endometrial samples was evaluated by immunohistochemistry. **b** Integrated optical density was analysed. ****P<0.0001. **c** Western blotting was performed to evaluate the protein expression of LPCAT1 at the translation level after transfection with a siRNA targeting LPCAT1 or a scrambled siRNA as a negative control (si-NC). **d** The relative proliferation ability of the transfected endometrial cancer cells was detected at a fixed time for 5days by the MTT assay. The growth curves were analysed using 2-way ANOVA. **e** Volcano plot of the DEGs generated by comparison of the high expression group and low expression group depending on the median expression of LPCAT1. Differentially expressed genes were determined by the Wilcoxon rank sum test with q=0.05 and log2FC transformation as the significance threshold. **f** GO and KEGG enrichment analyses of 378 DEGs. Terms with p and q<0.05 were considered to be significantly enriched. **g** Interaction network constructed with nodes with interaction confidence values>0.65

To verify LPCAT1 expression in EC, immunohistochemistry (IHC) was performed in EC tissue from 25 patients and 11 normal tissues. The results indicated that LPCAT1 expression in EC tissues was significantly higher than that in normal tissues (Fig.6a, b). Then, Ishikawa, RL95-2 and HEC-1A cells were transfected with si-LPCAT1 or si-NC. The Western blot results confirmed the decrease in the protein level of LPCAT1 (Fig.6c). Next, an MTT assay was performed to verify the effect of LPCAT1 silencing on EC cells. The results showed that LPCAT1 inhibited the proliferation of EC cells (Fig.6d). To determine the biological role of LPCAT1, we divided patients into the high expression group and low expression group according to the median expression of LPCAT1. DEGs were analysed by the limma package (Additional file 5: Table S3). The results are shown in volcano plots (Fig.6e), in which the red dots represent the upregulated genes screened on the basis of corrected P<0.05 and log_2_FC1, the green dots represent the downregulated genes screened on the basis of corrected P<0.05 and log_2_FC1, and the grey dots represent genes with no significant differences. A PPI network of the DEGs with confidence>0.65 was constructed (Fig.6g). Go enrichment analysis and KEGG pathway enrichment analysis of the DEGs were performed by R software (Fig.6f). The results of the GO analysis indicated that the DEGs were enriched in receptor ligand activity, endopeptidase inhibitor activity, hormone activity, transmembrane transporter complex, ion channel complex, motile cilium, pattern specification process, regionalization and anterior/posterior pattern specification. The KEGG results showed that salivary secretion was suggested to be an enriched pathway.

### Correlation of LPCAT1 expression with the proportion of TICs

**Fig. 7:**
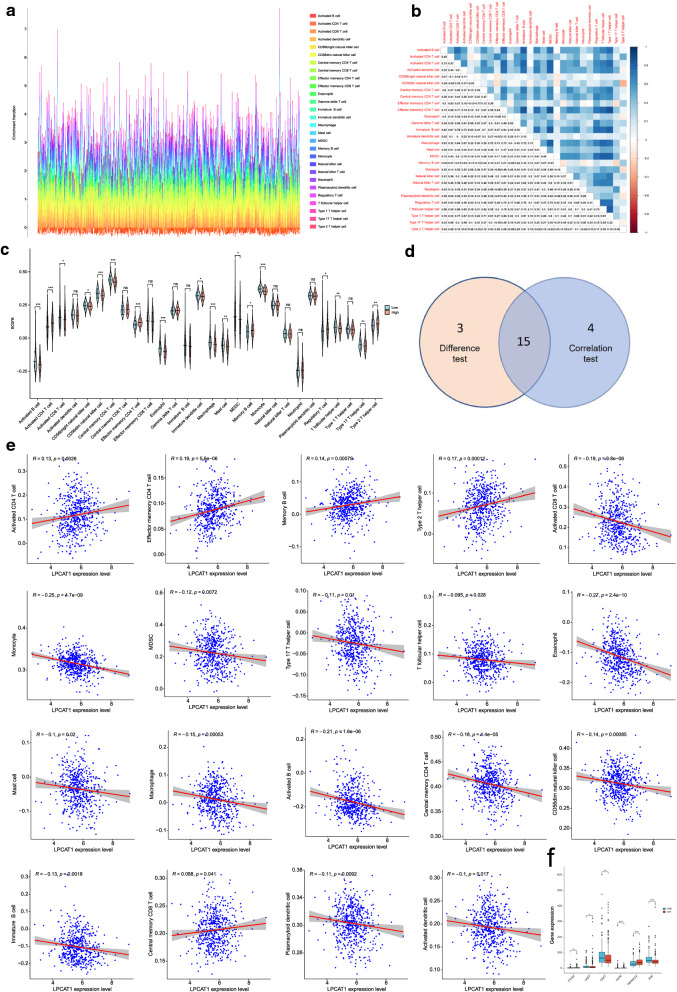
Correlation of the proportion of TICs in UCEC samples, and association of TICs proportion and typical ICGs with LPCAT1 expression. **a** Barplot showing the proportion of 22 types of TICs in the UCEC tumour samples. The column names of the barplot are the sample IDs. **b** Heatmap showing the correlation between 22 kinds of TICs, the p-value in each tiny box indicates the correlation between two cells. Persons correlation coefficient was used for statistical significance test. **c** Violin plot showing the differentiation ratio of 28 kinds of immune cells between UCEC tumour samples with high or low LPCAT1 expression relative to the median of LPCAT1 expression level, and the Wilcoxon rank sum was used as the statistical significance test. **d** Scatter plot showing the correlation of 19 TIC proportions with the LPCAT1 expression (p<0.05). The red line in each plot was the fitted curve of linear model indicating the immune cells proportion tropism along with LPCAT1 expression, and the Person correlation coefficient was applied for statistical significance test. **e** Venn plot displaying fifteen kinds of TICs correlated with LPCAT1 expression codetermined by correlation and difference tests displayed in violin and scatter plots, respectively. **f** Differential expression of ICGs between the LPCAT1 high-expression group and LPCAT1 low-expression group. *p<0.05, **p<0.01, ***p<0.001

We applied the ssGSEA algorithm to further confirm the correlation of LPCAT1 expression and immune component by analysing the proportion of tumour infiltrating immune cells and constructing 28 sorts of immune cell profiles for the UCEC samples (Fig.7a, b). By constructing difference and correlation analyses, 15 kinds of TICs were found to be significantly correlated with the expression of LPCAT1 (Fig.7ce). Among the TICs, 4 kinds of TICs were positively associated with LPCAT1 expression, including activated CD4 T cells, effector memory CD4 T cells, memory B cells and type 2T helper cells; 11 kinds of TICs were negatively correlated with LPCAT1 expression, including activated B cells, activated CD8 T cells, CD56dim natural killer cells, central memory CD4 T cells, effector memory CD8 T cells, eosinophils, macrophages, mast cells, MDSCs, monocytes, and T follicular helper cells. These results strongly indicated that LPCAT1 expression influenced the immune activity of the TME. Then, the correlation of LPCAT1 expression with immune checkpoint genes (ICGs) was determined to estimate the immunotherapy responses that involved LPCAT1 expression. The correlation between LPCAT1 expression and typical ICGs [cytotoxic T-lymphocyte associated protein 4 (CTLA4), lymphocyte activating 3 (LAG3), CD47, CD70, TNF receptor superfamily member 14 (TNFRSF14), CD155 (PVR), etc.] was determined, indicating that significantly different expression of ICGs was observed between the LPCAT1 high-expression group and LPCAT1 low expression group (Fig.7f). The results demonstrate that LPCAT1 may help evaluate immunotherapy responses in EC.

## Discussion

In the current study, we attempted to identify prognostic GPL-related genes involved in the tumour-microenvironment in endometrial cancer. LPCAT1 was confirmed to be associated with the immune activities. Importantly, ssGSEA indicated that LPCAT1 might serve as an indicator of TME status in UCEC patients.

Obesity, hypertension and diabetes are clearly recognized as risk factors for endometrial cancer. The metabolic changes of endometrial tumours compared with their nonmalignant counterparts have gradually been recognized. Studies have shown that glucose transport, which is mediated by GLUT-6 and glycolytic-lipogenic metabolism, may be responsible for tumour cell survival [19]. Significantly increased expression of SREBP1 and the subsequent enhancement of lipid synthesis are the main characteristics of EC [20]. A poor prognosis has been proven to be associated with the overexpression of FASN in endometrial cancer [21]. Although an increasing number of metabolic genes have been found to be responsible for endometrial cancer, there are few studies on glycerophospholipid-related metabolites. GPLs are necessary for cells to maintain homeostasis and normal physiological functions. Disorders of GPLs are obviously involved in benign disease and cancers [22,25]. The identification of dysregulated GPL-related genes may provide a novel perspective for EC therapy.

An increasing number of studies have shown that metabolic changes can affect the tumour microenvironment (TME), especially immune cells. The study of Ringel et al. demonstrated that free fatty acids (FFAs) were decreased in tumour microenvironment of mice fed a high-fat diet, and the exhaustion of free fatty acids not only inhibited the function of CD8+ T cells but also reduced their number [26]. TME components play an indispensable role in the initiation and development of tumorigenesis. Targeting TME remodelling may provide a potential therapeutic strategy to inhibit tumour progression. Several studies have demonstrated that the immune microenvironment influences tumour biological behaviour [9, 27, 28]. A lack of tumour-killing immune cells has been shown to be associated with the poor prognosis of various malignancies.

GPL metabolism influences the endometrial cancer TME. We systematically investigated the expression of 77 GPL-related genes in UCEC tumour tissues and their prognostic value. Twenty-three out of 77 genes were differentially expressed, and the expression of 11 genes was related to the prognosis of UCEC patients. These results indicated the potential role of GPL metabolism in EC and the possibility of targeting GPL-related genes as a treatment strategy. To identify core prognostic genes that play essential roles in EC, two machine learning algorithms and PPI analysis were used. Finally, we selected four significant genes. Then, patients were divided into the high-expression group and low-expression group according to the median expression of four significant genes, respectively. ImmuneScore, StromalScore and ESTIMATEScore were calculated by the estimate package in R to estimate the immune and stromal components of each patients. The higher ImmuneScore and the StromalScore, the larger the respective components in the TME. The scores were compared between the high-expression group and low-expression group and the results revealed that only LPCAT1 was related to the tumour microenvironment. The scores of LPCAT1 high expression were significantly lower than those of the LPCAT1 low expression group, suggesting that a high concentration of immune cells was found in the TME of the LPCAT1 low expression group. Here, we performed a transcriptomic analysis of UCEC in TCGA, which revealed that the increased expression of LPCAT1 was significantly associated with the advanced, specific subtypes and poor prognosis. Chen et al. demonstrated that the immune and stromal scores were positively correlated with the clinical outcomes of EC patients [10]. This might represent one of the mechanisms contributing to the better prognosis of the LPCAT1 low expression group. Accordingly, LPCAT1 may be a potential prognostic marker and a therapeutic target of the TME in UCEC.

LPCAT1 is a member of the lysophosphatidylcholine acyltransferase (LPCAT) family that regulates phospholipid metabolism in the Lands cycle. LPCAT1 catalyzes the transformation of lysophosphatidylcholine (LPC) into phosphatidylcholine (PC) by incorporating fatty acyl chains into phosphatidylcholine [29]. LPCAT1 was initially isolated from alveolar type II cells and is involved in the synthesis of alveolar surfactant [30]. Recently, LPCAT1 has been found to be overexpressed and to act as an oncogene in a variety of tumours [31]. However, no study has demonstrated the relationship of LPCAT1 in endometrial cancer and the tumour microenvironment.

To further investigate the functions of LPCAT1 in EC, an MTT assay was performed after silencing LPCAT1 in three EC cell lines. The result confirmed that LPCAT1 acted as an oncogene in EC. DEG analysis between the high LPCAT1 expression group and the low LPCAT1 expression group was performed, followed by GO and KEGG enrichment analyses. Our results showed that salivary secretion included enriched expression of DEGs according to KEGG analysis and receptor ligand activity, endopeptidase inhibitor activity, hormone activity, transmembrane transporter complex, ion channel complex, motile cilium, pattern specification process, regionalization, and anterior/posterior pattern specification included enriched expression of DEGs according to GO analysis. These results suggest that the underlying mechanism of LPCAT1 serves as a potential prognostic molecular marker and therapeutic target in EC.

Another important aspect of this study was the correlation between LPCAT1 expression and the level of immune infiltration, which is closely tied to the microenvironment of EC. Many studies have reported that various of immune cells, including different kinds of lymphocytes and macrophages, can form an immune microenvironment of EC, which consequently influences EC patient outcomes [32,34]. Thus, we analysed the relationship between LPCAT1 genomic alterations and immune infiltration in EC. Our results demonstrate that the upregulation of LPCAT1 was accompanied by increases in activated CD4 T cells, effector memory CD4 T cells, memory B cells and Type 2T helper cells, which were positively correlated with LPCAT1 expression. The upregulation of LPCAT1 was accompanied by reductions in activated B cells, activated CD8 T cells, CD56dim natural killer cells, central memory CD4 T cells, effector memory CD8 T cells, eosinophils, macrophages, mast cells, MDSCs, monocytes, T follicular helper cells which were negatively correlated with the expression of LPCAT1. Meanwhile, LPCAT1 might act as a predictor for estimating immunotherapy responses. Together these findings suggested that the LPCAT1 played an important role in the recruitment and regulation of immune infiltrating cells in UCEC.

However, the lack of experimental proof is a limitation of this study. Although the IHC assay suggested the LPCAT1 expression was significantly higher in EC than in normal tissues and the MTT assay proved that LPCAT1 acted as an oncogene, the potential role of LPCAT1 and its relationship with immune cells is not sufficient without further support from in vivo and in vitro experiments. A better understanding of the functions and underlying mechanism of LPCAT1 in EC will be achieved by further verification experiments and clinical trials which might improve the accuracy of diagnosis and therapy strategies. Therefore, further studies should be conducted to verify the accuracy of the combined analysis of LPCAT1 expression, GPLs and the amounts of tumour-infiltrating immune cells in UCEC patients (Additional file 4: Tables S2; Additional file 5: Tables S3).

## Conclusion

Our findings provide specific insight into the role of GPL-related genes in EC. We concluded that LPCAT1 might serve as a molecular marker to predict the prognosis and status of the EC microenvironment. The results of this study further suggested the potential mechanism of LPCAT1 as a novel therapeutic target for improving clinical outcomes. We strongly recommend further investigation of this topic to clarify exact biological impact of LPCAT1.

## Supplementary Information

**Additional file 1: Figure S1.** (a) ROC (receiver operating characteristic) curve and (b) contribution of the risk score in patients who died and survived.

**Additional file 2: Figure S2.** (a) Contribution of the predictive value of patients in the training set. (b) Contribution of the predictive value of patients in the testing set.

**Additional file 3: Table S1.** Immune scores and stromal scores of EC.

**Additional file 4: Table S2.** Clinical data of UCEC patients.

**Additional file 5: Table S3.** Differentially expressed genes selected based on the median expression of LPCAT1.

## Data Availability

The datasets generated and analyzed during this study are available in the TCGA database (https://portal.gdc.cancer.gov) and GEO database (https://www.ncbi.nlm.nih.gov/geo/).

## Abbreviations

EC: Endometrial cancer
TCGA: The Cancer Genome Atlas
UCEC: Uterine corpus endometrial carcinoma
GPLs: Glycerophospholipids
TME: Tumor microenvironment
MSigDB: Molecular signatures database
LASSO: Least absolute shrinkage and selection operator
PPI: Proteinprotein intersection
IHC: Immunohistochemistry
GO: Gene ontology
KEGG: Kyoto Encyclopedia of Genes and Genomes
ssGSEA: Single sample geneset enrichment analysis
LPCAT1: Lysophosphatidylcholine acyltransferase1
TICs: Tumor infiltrating cells
PLA2s: Phospholipase A2 enzymes
LPCAT3: Lysophosphatidylcholine acyltransferase 3
CHKA: Choline kinase
ECM: Extracellular matrix
DEGs: Differential expression genes
OS: Overall survival
GEO: Gene expression omnibus
DFI: Disease free interval
PFS: Progress free survival
ROC: Receiver operating characteristic
AUC: Areas under ROC curve
MDSC: Myeloid-derived suppressor cells
