# Supplementary figures and images for: Elevated expression of LPCAT1 predicts a poor prognosis and is correlated with the tumour microenvironment in endometrial cancer
Source: Cancer Cell Int. 2021 May 20;21:269. doi: 10.1186/s12935-021-01965-1 (PMC8139085; doi:10.1186/s12935-021-01965-1)

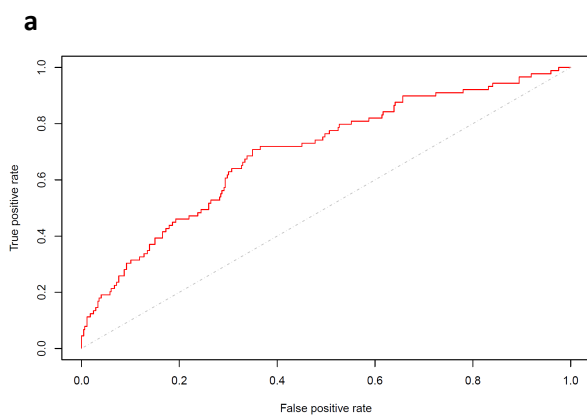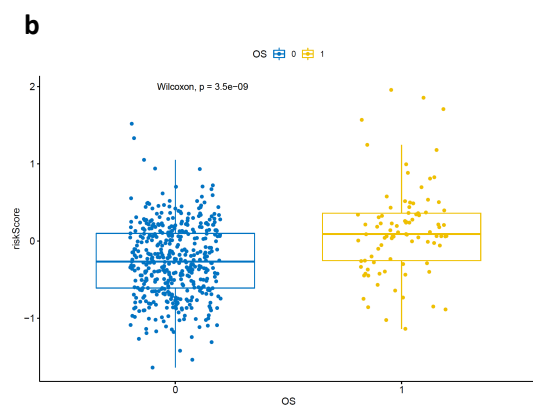

Supplement: Supplementary file 1 — Additional file 1: Figure S1. (a) ROC (receiver operating characteristic) curve and (b) contribution of the risk score in patients who died and survived. [file 12935_2021_1965_MOESM1_ESM.pdf]

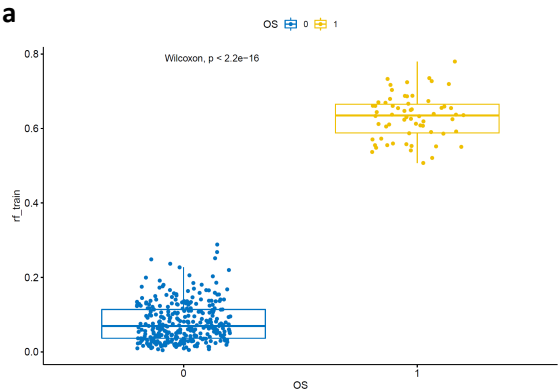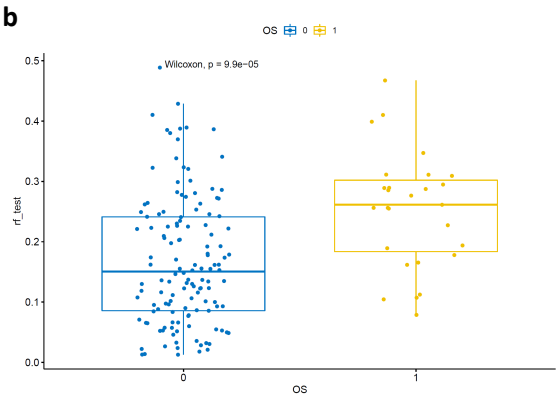

Supplement: Supplementary file 2 — Additional file 2: Figure S2. (a) Contribution of the predictive value of patients in the training set. (b) Contribution of the predictive value of patients in the testing set. [file 12935_2021_1965_MOESM2_ESM.pdf]
